# The molecular mechanism underlying mitophagy‐mediated hippocampal neuron apoptosis in diabetes‐related depression

**DOI:** 10.1111/jcmm.16763

**Published:** 2021-07-02

**Authors:** Jian Liu, Lin Liu, Yuan‐Shan Han, Jian Yi, Chun Guo, Hong‐Qing Zhao, Jia Ling, Yu‐Hong Wang

**Affiliations:** ^1^ The First Hospital Hunan University of Chinese Medicine Hunan Province Changsha China; ^2^ Institute of Innovation and Applied Research Hunan University of Chinese Medicine Hunan Province Changsha China; ^3^ Key Laboratory of Chinese Material Medical Power and Innovation Drugs Established by Provincial and Ministry Hunan University of Chinese Medicine Hunan Province Changsha China

**Keywords:** Apoptosis, Diabetes‐related depression, GluR2, Glutamate, Hippocampal neuron, Mitophagy, Parkin

## Abstract

Diabetes‐related depression (DD) is a major complication of diabetes mellitus. Our previous studies indicated that glutamate (Glu) and hippocampal neuron apoptosis are key signal and direct factor leading to diabetes‐related depression, respectively. However, the accurate pathogenesis remains to be unclear. We hypothesized that diabetes‐related depression might be associated with the mitophagy‐mediated hippocampal neuron apoptosis, triggered by aberrant Glu‐glutamate receptor2 (GluR2)‐Parkin pathway. To testify this hypothesis, here the rat model of DD in vivo and in vitro were both established so as to uncover the potential mechanism of DD based on mitophagy and apoptosis. We found that DD rats exhibit an elevated glutamate levels followed by monoamine neurotransmitter deficiency and depressive‐like behaviour, and DD modelling promoted autophagosome formation and caused mitochondrial impairment, eventually leading to hippocampal neuron apoptosis via aberrant Glu‐GluR2‐Parkin pathway. Further, in vitro study demonstrated that the simulated DD conditions resulted in an abnormal glutamate and monoamine neurotransmitter levels followed by autophagic flux increment, mitochondrial membrane potential reduction and mitochondrial reactive oxygen species and lactic dehydrogenase elevation. Interestingly, both GluR2 and mammalian target of rapamycin (mTOR) receptor blocker aggravated mitophagy‐induced hippocampal neuron apoptosis and abnormal expression of apoptotic protein. In contrast, both GluR2 and mTOR receptor agonist ameliorated those apoptosis in simulated DD conditions. Our findings revealed that mitophagy‐mediated hippocampal neuron apoptosis, triggered by aberrant Glu‐GluR2‐Parkin pathway, is responsible for depressive‐like behaviour and monoamine neurotransmitter deficiency in DD rats. This work provides promising molecular targets and strategy for the treatment of DD.

## 1 INTRODUCTION

Diabetes mellitus is a devastating chronic metabolic disease with numerous complications, such as diabetic nephropathy, diabetic retinopathy and diabetes‐related depression (DD).^1^, ^2^ According to World Health Organization, more than 463 million people worldwide suffer from diabetes mellitus in 2019, and the number of patients will increase to 702 million by 2045. Additionally, people with diabetes mellitus have a high risk of depressive symptoms, which is higher than that observed in non‐diabetics.^3^, ^4^ Moreover, morbidity and suicide rates of DD patients are increasing per year.^5^, ^6^ Thus, revealing the pathogenesis of DD is considered an urgent need.

Emerging evidence indicated that excitatory neural toxicity of glutamate, monoamine neurotransmitter deficiency, autophagy and apoptosis are all involved in the onset of depression.^7^, ^8^ Hippocampus is recognized as a vital tissue that is not only affected by diabetes mellitus but is also linked to depression.^9^ So, do these causes of depression also lead to DD? Interestingly, our previous studies found that glutamate and hippocampal neuron apoptosis are key signals and direct factors leading to diabetes‐related depression, respectively.^10^, ^11^ However, further research remains to be needed to elucidate the exact mechanism of DD based on apoptosis.

Autophagy, including mitophagy, is a tightly modulated cellular degradation process responsible for the clearance of damaged proteins and organelles.^12^ Generally, excessive mitophagy brings about mitochondrial impairment and further triggers hippocampal neuron apoptosis via the death receptor pathway of endogenous.^13^ An increasing number of studies also indicate the relationship between autophagy and apoptosis.^14^, ^15^ It has been accepted that increased hippocampal neuron apoptosis plays an important role in the pathogenesis of diabetes‐related depression.^10^, ^16^ In addition, various evidence suggested that abnormally elevated glutamate levels had a particularly negative effect on memory, due to the excessive release of excitatory amino acids and the activation of their receptors.^17^, ^18^ Notwithstanding our preliminary studies have also demonstrated that behavioural or morphological, glutamate and neurotransmitter changes in DD rats are similar to those observed in vitro simulated DD conditions and indicated that the hippocampal neuron autophagy which is activated by glutamate receptor2 (GluR2)/mammalian target of rapamycin (mTOR) signal pathway is of great importance for pathogenesis of diabetes‐related depression.^19^, ^20^ However, the explicit pathway that how does excessive glutamate activates autophagy and hippocampal neuron apoptosis remains to be uncovered.

Therefore, we hypothesized that diabetes‐related depression might be associated with the mitophagy‐mediated hippocampal neuron apoptosis, triggered by aberrant Glu‐GluR2‐Parkin pathway. In the present study, we prepare rat model of DD in vivo and in vitro, detect depressive‐like behaviour, neurotransmitter, autophagosome, autophagy flux, mitochondrial function and apoptosis, and further use antagonists and agonists for key signalling pathway proteins so as to test this hypothesis.

## 2 MATERIALS AND METHODS

### 2.1 Animals

Specific‐pathogen‐free adult male Sprague‐Dawley rats (weight 200‐220g) and pregnant female Sprague‐Dawley rats (E16‐18 embryos) were provided by Hunan Slac Jingda Laboratory Animal Co., Ltd., Changsha, China (license No. SCXK (Xiang) 2019‐0004). All rats were allowed free access to food and water. All experiments were approved by the Laboratory Animal Ethics Committee at the First Hospital of Hunan University of Chinese Medicine (approval number: HN‐ZYFY‐2020‐01‐02).

### 2.2 Modelling of DD Rats and Pharmacological Interventions

The model of DD rats was established as described in our previous study.^10^, ^21^ In brief, the experimental model of diabetes mellitus (DM) was induced with a combination of low‐dose STZ and a high‐fat diet (HFD). Firstly, the rats were fed with a HFD for two weeks and then intraperitoneally received 38 mg/kg STZ (Solarbio) after fasting overnight. Fasting blood glucose was tested three days after STZ injection. Rat with blood glucose over 16.70 mmol/L was considered diabetic and selected for the subsequent study. Afterwards, diabetic rats were exposed to 28 days of chronic unpredictable mild stress (CUMS) to establish a model of DD rats according to the methods of Willner (year) with modifications and our previous studies.^9^ During a period of CUMS, meanwhile, rats were given an intraventricular administration with GluR2 receptor blocker CNQX (MCE) and its agonist CI‐HIBO (APExBIO), and mTOR receptor blocker rapamycin (MCE) and its agonist MHY1485 (MCE), respectively.

### 2.3 Open‐Field Test

Open‐field test was used to evaluate the spontaneous locomotor activity and exploratory behaviours. Briefly, an 80 cm × 80 cm × 40 cm open field was utilized in this experiment. The bottom of the box was divided into 25 equilateral squares. The rats were placed in the centre of the open field, after that the horizontal movements (four feet within a square counted as one score) and vertical movements were counted by scoring within three minutes after one‐minute adaptation.

### 2.4 Forced Swim Test

Forced swim test was performed for assessing giving up‐like depression behaviour. Briefly, this test needs a circular fibreglass pool containing warm water (25 ± 1℃). And in this experiment, all rats were given one minute to adapt and three minutes to swim. Immobility time was determined by the time a rat stopped struggling. Moreover, moved slowly to remain floating in the water was seen as immobility.

### 2.5 Modelling of DD Hippocampal Neurons and Pharmacological Interventions

Hippocampal neurons from SD rats were isolated, purified and cultured. Then, DD conditions (ie cell model of DD) were simulated according to our previous study.^11^, ^16^ Briefly, after hippocampal neurons were primarily cultured for 5‐7 days, 150 mM glucose (Solarbio, China) and 200 μM corticosterone (Solarbio, China) were added to the cell wells for 18 hours to establish an in vitro DD model according to our previous studies.^11^, ^16^ After DD modelling, meanwhile, cells were treated with GluR2 receptor blocker CNQX and its agonist CI‐HIBO, and mTOR receptor blocker rapamycin its agonist MHY1485, individually, for 18 hours.

### 2.6 Enzyme‐Linked Immunosorbent Assay

The homogenate from hippocampus and the supernatant from hippocampal neuron cells were collected after the establishment of the DD model in vivo and in vitro, respectively. The levels of Glu, 5‐HT and DA were measured by using an enzyme‐linked immunosorbent assay kit (R&D, USA).

### 2.7 Tunel Staining

After DD model establishment and treatment with CNQX, CI‐HIBO, rapamycin or MHY1485 for 18 hours, hippocampal neurons were fixed with 4% paraformaldehyde for 30 minutes and treated with 0.2% Triton X‐100 for 5 minutes and treated with 0.3% H_2_O_2_ for 2 minutes. Then, cells were incubated with terminal deoxynucleotidyl transferase (TdT)‐mediated dUTP nick end labelling (TUNEL) reaction mix (Roche, Switzerland) for 60 minutes at 37℃ in the dark. Apoptosis among hippocampal neurons was observed by laser scanning confocal microscope (Zeiss).

### 2.8 mRFP‐GFP‐LC3 transfection and Autophagic Flux Detection

Primary hippocampal neurons were seeded in 24‐well plates and cultured for 5‐7 days. After DD model establishment and treatment with CNQX or CI‐HIBO for 18 hours, cells were transfected with 1 × 10^9^ PFU/ml autophagy double‐standard adenovirus (mRFP‐GFP‐LC3) and manipulated in accordance with the instruction of mRFP‐GFP‐LC3 (HANBIO, China). Six hours after transfection, reagents were replaced by Neurobasal medium (Gibco) containing 2% B27 (Gibco) and cultured overnight. Afterwards, cells were fixed with 4% paraformaldehyde for 30 minutes and then incubated with 4′,6‐diamidino‐2‐phenylindole (DAPI) (1:800, ab228549, Abcam, USA) at room temperature for 15 minutes. Autophagosome, autolysosome and autophagic flux were observed by a laser scanning confocal microscope.

### 2.9 Immunofluorescent Staining

After treated with CNQX, CI‐HIBO, rapamycin or MHY1485 for 18 hours, hippocampal neurons were fixed with 4% paraformaldehyde for 30 minutes and then treated with 0.25% Triton X‐100 for 15 minutes and blocked in 5% bovine serum albumin for 30 minutes, before incubation with primary antibodies, overnight, in the dark, in a humidified container, at 4℃. The following antibodies were used: rabbit anti‐Parkin polyclonal antibody (1:100, 14060‐1‐AP, Proteintech), rabbit anti‐GluR2 polyclonal antibody (1:100, 11994‐1‐AP, Proteintech), rabbit anti‐mTOR polyclonal antibody (1:100, 20657‐1‐AP, Proteintech), rabbit anti‐Beclin‐1 polyclonal antibody (1:100, ab210498, Proteintech), rabbit anti‐Cyt‐c polyclonal antibody (1:100, 10993‐1‐AP, Proteintech), rabbit anti‐Bax polyclonal antibody (1:100, 50599‐2‐Ig, Proteintech), rabbit anti‐caspase‐9 polyclonal antibody (1:100, 10380‐1‐AP, Proteintech). The hippocampal neurons were then incubated with anti‐rabbit IgG, conjugated to fluorescein isothiocyanate (1:400, SA00003‐2, Proteintech) at 37℃ for 30 minutes and stained with DAPI (1:800, Abcam) at room temperature for 15 minutes. Finally, a laser scanning confocal microscope was used to analyse protein expression.

### 2.10 Mitochondrial Membrane Potential Detection

After treated with CNQX, CI‐HIBO, rapamycin or MHY1485 for 18 hours, hippocampal neurons were incubated with 10 μg/ml JC‐1 fluorescent probe (Beyotime, China) at 37℃ for 20 minutes. Afterwards, mitochondrial membrane potential (MMP) was measured at 585 nm and 514 nm, using a laser scanning confocal microscope.

### 2.11 Mitochondrial Reactive Oxygen Species (ROS) detection

After treated with CNQX, CI‐HIBO, rapamycin or MHY1485 for 18 hours, hippocampal neurons were incubated with 5 μM MitoSOX fluorescent probe (Invitrogen) at 37℃ for 20 minutes. Then, cells were stained with DAPI (1:800, Abcam) at room temperature for 15 minutes. Finally, mitochondrial reactive oxygen species (ROS) was analysed by a laser scanning confocal microscope.

### 2.12 Transmission Electron Microscopic (TEM) Analysis

Transmission electron microscopy was used to evaluate mitochondrial impairment and autophagosome formation in hippocampal neuron of DD rats. Briefly, after DD model establishment and treatment with CNQX, CI‐HIBO, rapamycin or MHY1485 for 21 days, hippocampus of each group rat was fixed with 1% osmium tetroxide, stained in aqueous uranyl acetate and then dehydrated and embedded in epoxy resin. Afterwards, sections were stained by using lead citrate and examined with a transmission electron microscope (Hitachi, Japan).

### 2.13 Western Blot Analysis

Western blot assay was used to detect the expression of Glu‐GluR2‐Parkin pathway protein and the endogenous apoptosis protein of Cyt‐c in hippocampal tissue of rats. The total protein content was detected by bicinchoninic acid assay (BCA). Proteins were separated on polyacrylamide gels and transferred to polyvinylidene fluoride membranes. Membranes were blocked in skimmed milk and probed with rabbit anti‐GluR2 (1:1000, 11994‐1‐AP, Proteintech), rabbit anti‐P62 (1:1000, 18420‐1‐AP, Proteintech), rabbit anti‐Parkin (1:1000, 14060‐1‐AP, Proteintech), rabbit anti‐Cyt‐c (1:1000, 10993‐1‐AP, Proteintech) and rabbit anti‐β‐actin (1:3000, 20536‐1‐AP, Proteintech), at 4℃ overnight. The blots were washed twice with tris‐buffered saline before incubation with anti‐rabbit secondary antibody horseradish peroxidase (1:400, BM5180, Boster), at room temperature for one hour. Finally, the expression levels of apoptosis‐associated proteins were quantified, using AlphaEase FC (Alpha Innotech, San Leandro, CA).

### 2.14 Statistics

Data are expressed as the mean ±standard deviation (SD) and calculated for each test group. All data were analysed by SPSS 16.0 (SPSS). A one‐way analysis of variance, followed by Dunnett's post hoc test, was performed for comparisons among groups. *P* < .05 was considered significant.

## 3 RESULT

### 3.1 DD rats exhibit an abnormal glutamate level elevation and depressive‐like behaviour

**FIGURE 1:**
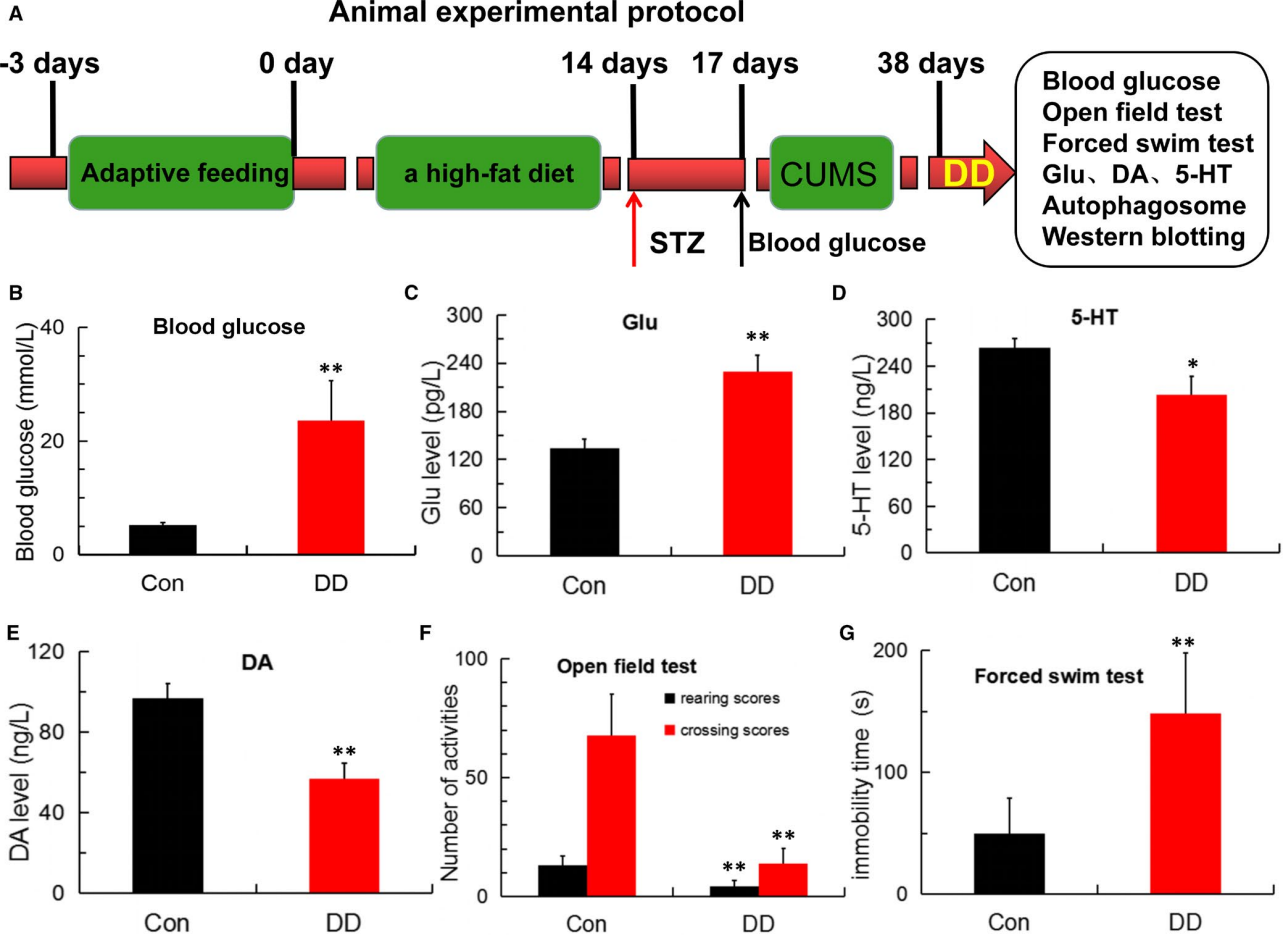
Abnormal Glu, 5‐HT, DA levels and depressive‐like behaviour in DD rats. (A) Experimental protocol. Rats were adaptively fed for 3 days then given to a high‐fat diet for 2 weeks and injected with STZ and followed by subjected CUMS for 21 days to establish the DD model. (B) Blood‐glucose levels detected by a blood‐glucose sensor in control and DD model group (n = 5; **P < .001 vs Con). (C) Glutamate content detected by ELISA in control and DD model group (n = 4; **P < .001 vs Con). (D‐E) 5‐HT and DA levels detected by ELISA in control and DD model group (n = 4; *P < .05, **P < .001 vs Con). (F‐G) Number of activities in the open‐field test and the immobility time in the forced swimming test (n = 5; **P < .001 vs Con)

As shown in Figure 1A, SD rats were given to high‐fat diet for 2 weeks and then injected with STZ and followed by subjected CUMS for 21 days to establish the diabetes‐related depression model. After modelling, we found that blood‐glucose level was obviously increased and glutamate level in hippocampus was significantly raised compared with those in control rats(Figure 1B‐C), and the levels of hippocampal monoamine neurotransmitter for 5‐HT and DA in model were dramatically decreased in comparison with control group(Figure 1D‐E). Furthermore, DD modelling obviously reduced the crossing scores and rearing scores in the open‐field test and also significantly increased the immobility time of DD rats in the forced swimming test(Figure 1F‐g). These results indicated that the rat model of DD was successfully established and the DD rats exhibit depressive‐like behaviour, together with abnormal glutamate level elevation and monoamine neurotransmitter deficiency.

### 3.2 Simulated DD conditions in vitro lead to hippocampal neurons apoptosis with abnormal levels of glutamate and monoamine neurotransmitter

**FIGURE 2:**
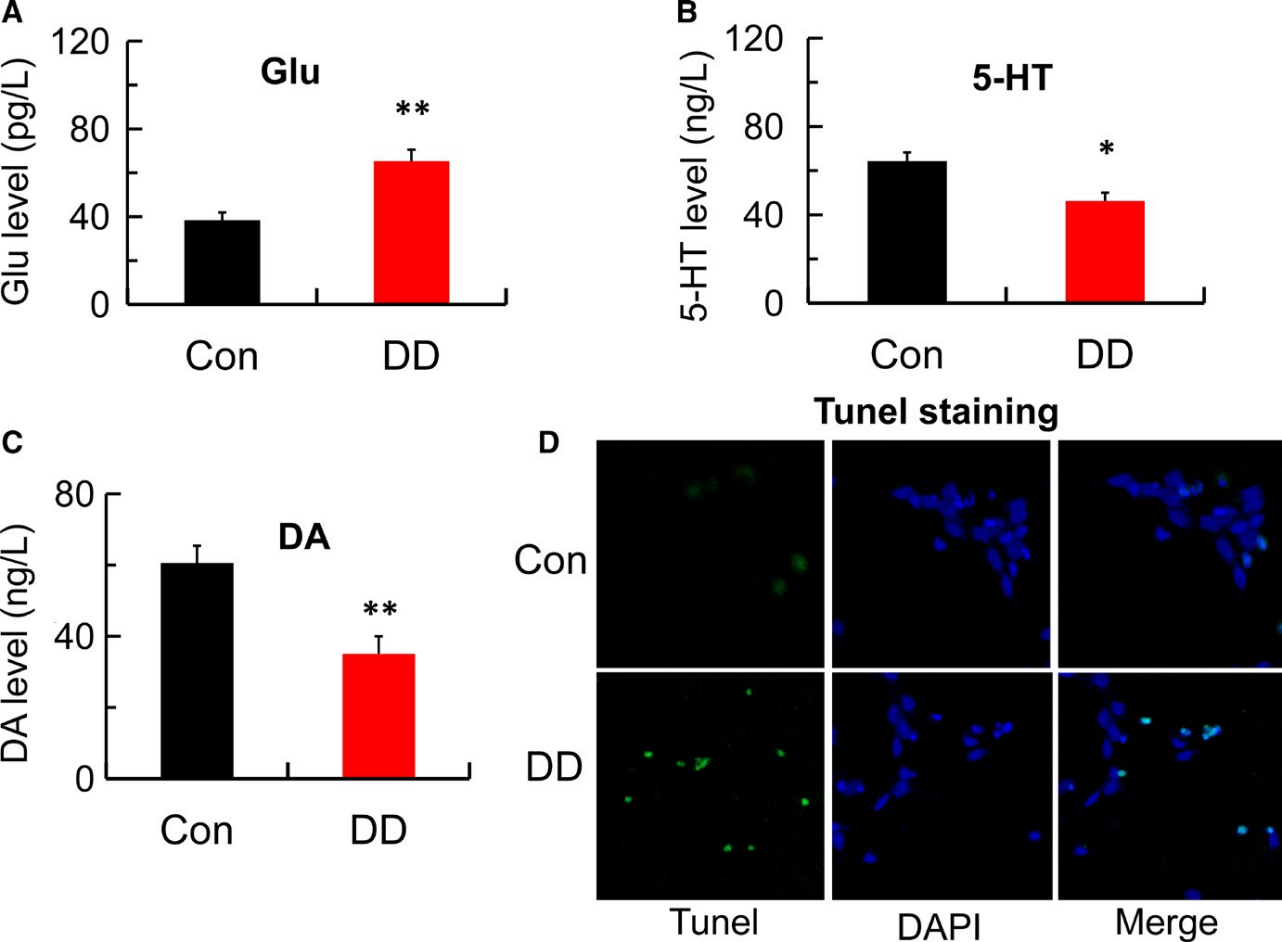
Abnormal Glu, 5‐HT, DA levels and hippocampal neurons apoptosis in simulated DD conditions. (A) Glutamate content detected by ELISA in control and DD model group (n = 4; **P < .001 vs Con). (B‐C) 5‐HT and DA levels detected by ELISA in control and DD model group (n = 4; *P < .05, **P < .001 vs Con). (D) Hippocampal neurons apoptosis detected by Tunel staining in control and DD model group (magnification:200×)

Figure 2A shows that glutamate level in simulated DD conditions group was significantly higher than those in the control group. The levels of monoamine neurotransmitter for 5‐HT and DA under simulated DD conditions were markedly decreased compared with that in the corresponding control group(Figure 2B‐C). Furthermore, as shown in Figure 2D, apoptosis among hippocampal neurons under simulated DD conditions was increased compared with control neurons. Therefore, we can conclude that the simulated DD conditions also increased the levels of glutamate and decreased the levels of 5‐HT and DA, which may be associated with the hippocampal neurons apoptosis, one of the important mechanisms of diabetes‐related depression.

### 3.3 Glu‐GluR2 signal triggers hippocampal neurons mitophagy under simulated DD conditions in vitro

**FIGURE 3:**
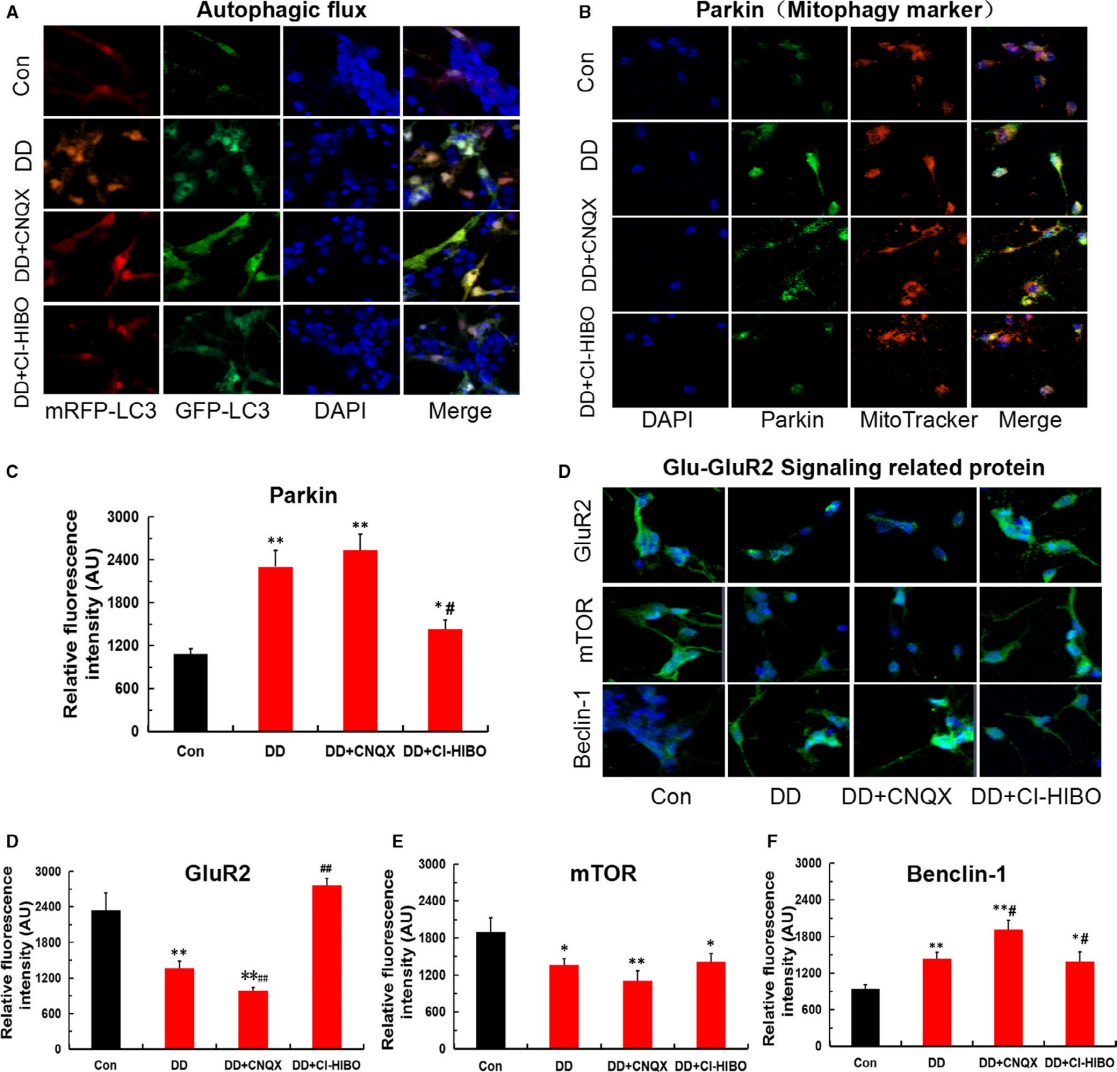
The simulated DD conditions lead to hippocampal neurons mitophagy via Glu‐GluR2 signal. (A) Autophagic flux was monitored by confocal microscopy and mRFP‐GFP‐LC3 virus transfection. (B‐C) Mitophagy marker protein of Parkin detected by immunofluorescence staining in each group (magnification:200×; n = 3; *P < .05, **P < .001 vs Con; ^#^ *P* < .05 vs DD). (D‐G) Glu‐GluR2 signal‐related proteins of GluR2, mTOR and Beclin‐1 detected by immunofluorescence staining in each group (magnification:200×; n = 3; *P < .05, **P < .001vs Con; ^#^ *P* < .05 vs DD, ^##^ *P* < .01 vs DD)

To investigate how elevated glutamate leads to hippocampal neurons apoptosis and to clarify the potential mechanism, we used immunofluorescence analysis to observe autophagy flux and detected the Glu‐GluR2 pathway‐related proteins in hippocampal neurons. As shown in Figure 3A, compared with control, the simulated DD conditions enhanced GFP‐LC3 and mRFP‐LC3 expression, suggesting it can both initiate autophagosomal formation and promote autophagy flux. Furthermore, GluR2 receptor blocker (CNQX) aggravated the hippocampal neurons autophagy flux formation. However, abnormal high levels of autophagy flow stimulated by simulated DD conditions were reversed by GluR2 receptor agonist (CI‐HIBO) treatment. Additionally, simulated DD conditions caused the obvious down‐regulation of GluR2 and mTOR immunopositivity and the up‐regulation of mitophagy‐associated Parkin and Beclin‐1 immunopositivity compared with the control conditions in hippocampal neurons. Moreover, CNQX exacerbated the disordered GluR2, mTOR, Parkin (a mitophagy marker protein) and Beclin‐1 immunopositivity. In contrast, abnormal expression of those proteins was significantly reversed by CI‐HIBO(Figure 3B‐g). Accordingly, these findings suggested that simulated DD conditions stimulated the mitophagy of hippocampal neurons, induced by Glu‐GluR2 signal, which may be associated with hippocampal neurons apoptosis.

### 3.4 Mitophagy causes mitochondrial dysfunction and further hippocampal neurons apoptosis under simulated DD conditions in vitro

**FIGURE 4:**
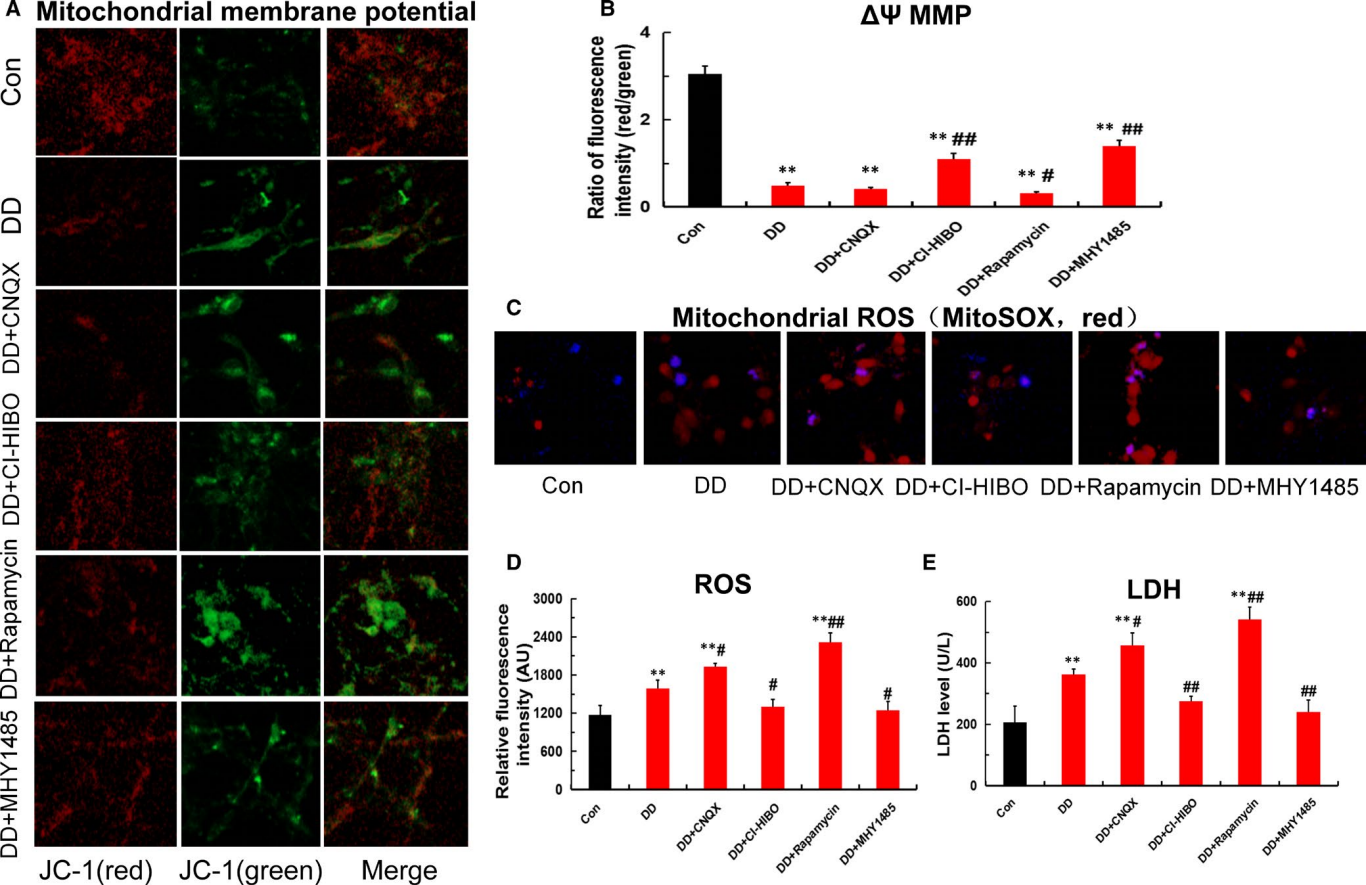
Mitochondrial dysfunction in hippocampal neurons under the simulated DD conditions. (A‐B) Mitochondrial membrane potential (MMP) detected by JC‐1 fluorescent probe in each group(magnification:200×; n = 3; **P < .001 vs Con; ^#^ *P* < .05, ^##^ *P* < .01 vs DD). (C‐D) Mitochondrial ROS detected by MitoSOX probe in each group (magnification:200×; n = 3; **P < .001 vs Con; ^#^ *P* < .05, ^##^ *P* < .01 vs DD). (E) LDH detected by ELISA in each group (n = 3; **P < .001vs Con; ^#^ *P* < .05, ^##^ *P* < .01 vs DD)

**FIGURE 5:**
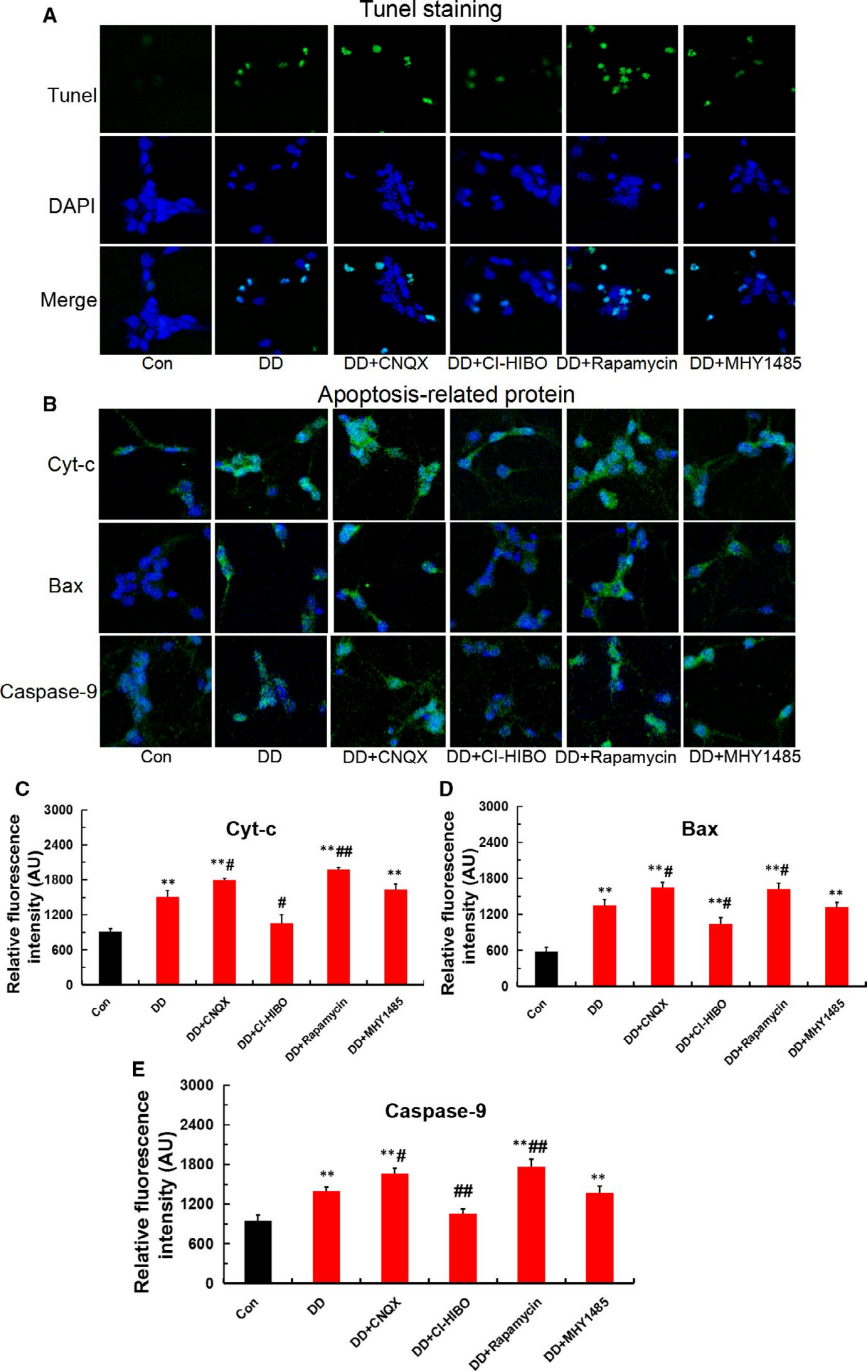
Mitophagy‐induced hippocampal neurons apoptosis under the simulated DD conditions. (A) Hippocampal neurons apoptosis detected by Tunel staining in each group(magnification:200×). (B‐E) Apoptotic proteins of Cyt‐c, Bax and caspase‐9 detected by immunofluorescence staining in each group (magnification:200×; n = 3; **P <.001 vs Con; ^#^ *P* < .05, ^##^ *P* < .01 vs DD)

The above works revealed that the Glu‐GluR2‐Parkin signal pathway given rise to mitophagy. It is by now generally accepted autophagy and apoptosis are closely associated. To confirm the effect of mitophagy on apoptosis, in this paper mitochondrial function and apoptosis were tested in vitro hippocampal neurons. As shown in Figure 4A‐B, mitochondrial membrane potential in DD group was remarkably decreased, and mitochondrial ROS and LDH were obviously raised compared with that of control. Simultaneously, GluR2 receptor blocker (CNQX) and mTOR receptor blocker (rapamycin) worsen the decline of mitochondrial membrane potential and the increment of mitochondrial ROS and LDH, whereas those disorders was ameliorated by GluR2 receptor agonist (CI‐HIBO) and mTOR receptor agonist (MHY1485)(Figure 4C‐E). Furthermore, Tunel staining and immunofluorescence staining results indicated that the simulated DD conditions enhanced apoptosis(Figure 5A) and apoptosis‐related proteins of Cyt‐c, Bax and caspase‐9 expression(Figure 5B‐E). Moreover, both CNQX and Rapamycin aggravated the mitophagy‐induced hippocampal neurons apoptosis. In contrast, the up‐regulation of apoptotic proteins was remarkably ameliorated after CI‐HIBO and MHY1485 intervention. In short, these results indicated that mitophagy led to mitochondrial dysfunction, which was responsible for hippocampal neurons apoptosis via the death receptor pathway of endogenous.

### 3.5 Aberrant Glu‐GluR2‐Parkin pathway leads to monoamine neurotransmitter deficiency and depressive‐like behaviour in DD rats

**FIGURE 6:**
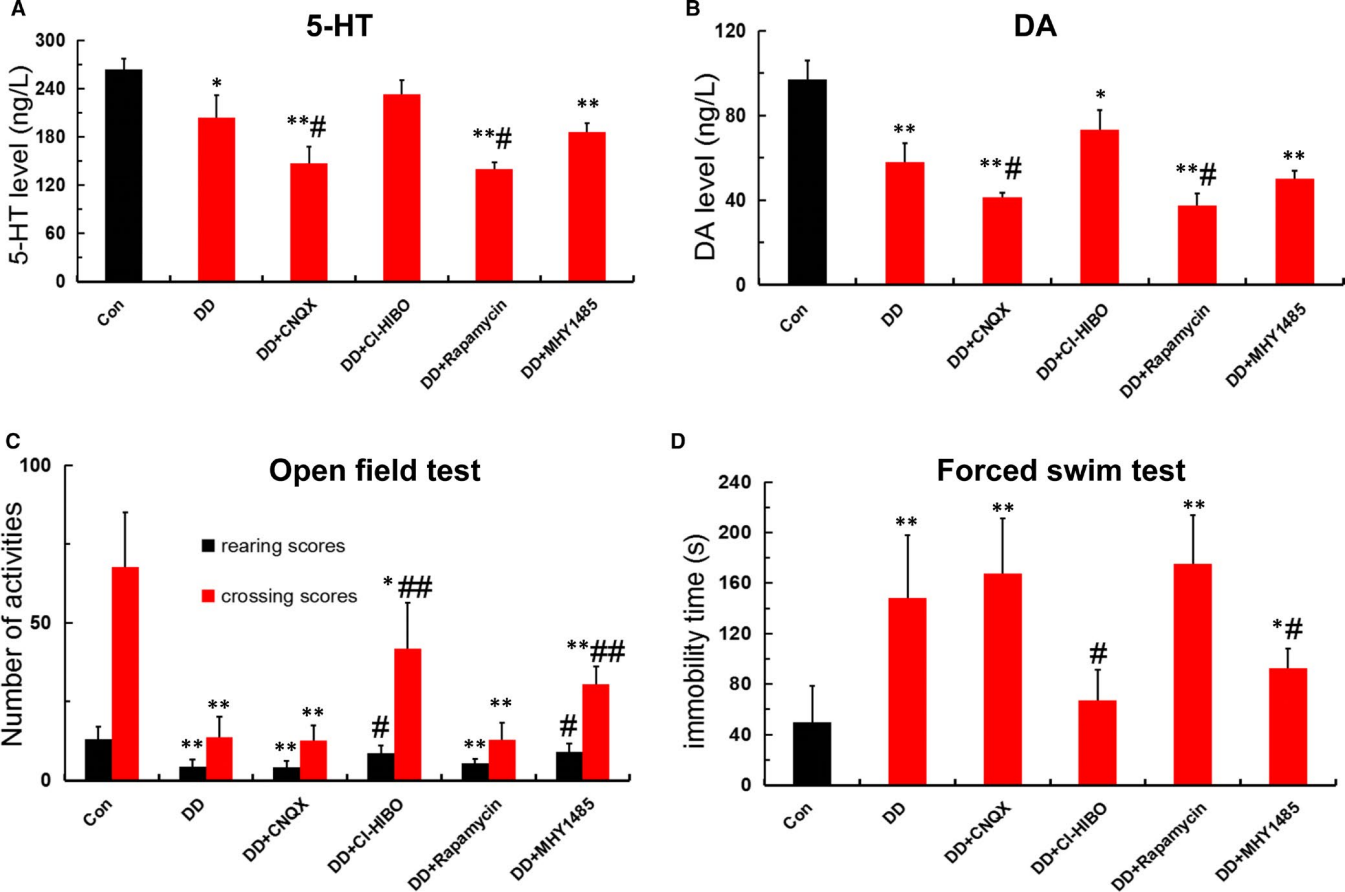
Aberrant Glu‐GluR2‐Parkin pathway leads to monoamine neurotransmitter deficiency and depressive‐like behaviour in DD rats. (A‐B) 5‐HT and DA levels detected by ELISA in each group (n = 4; *P < .05,**P < .001vs Con; ^#^ *P* < .05, ^##^ *P* < .01 vs DD). (C‐D)Number of activities in open‐field test and the immobility time in forced swimming test among each group (n = 5; *P < .05, **P < .001 vs Con; ^#^ *P* < .05 vs DD)

To test whether the Glu‐GluR2‐Parkin pathway also changes the neurotransmitter levels and depressive‐like behaviour in DD rats, model rats were treated with GluR2 receptor blocker CNQX, GluR2 receptor agonist CI‐HIBO, mTOR receptor blocker rapamycin and mTOR receptor agonist MHY1485, respectively. The results show that both CNQX and rapamycin aggravated the monoamine neurotransmitter of 5‐HT and DA deficiency(Figure 6A‐B), reducing the crossing scores and rearing scores in open‐field test and increasing the immobility time of DD rats in forced swimming test when compared with those in DD rats(Figure 6C‐D). In contrast, both CI‐HIBO and MHY1485 alleviated the abnormal monoamine neurotransmitter levels and depressive‐like behaviour in DD rats. Thus, these findings manifested that Glu‐GluR2‐Parkin pathway was involved in the monoamine neurotransmitter deficiency and depressive‐like behaviour in DD rats.

### 3.6 Aberrant Glu‐GluR2‐Parkin pathway triggered depression‐like behaviour in DD rats was relevant to mitophagy‐mediated hippocampal neurons apoptosis

**FIGURE 7:**
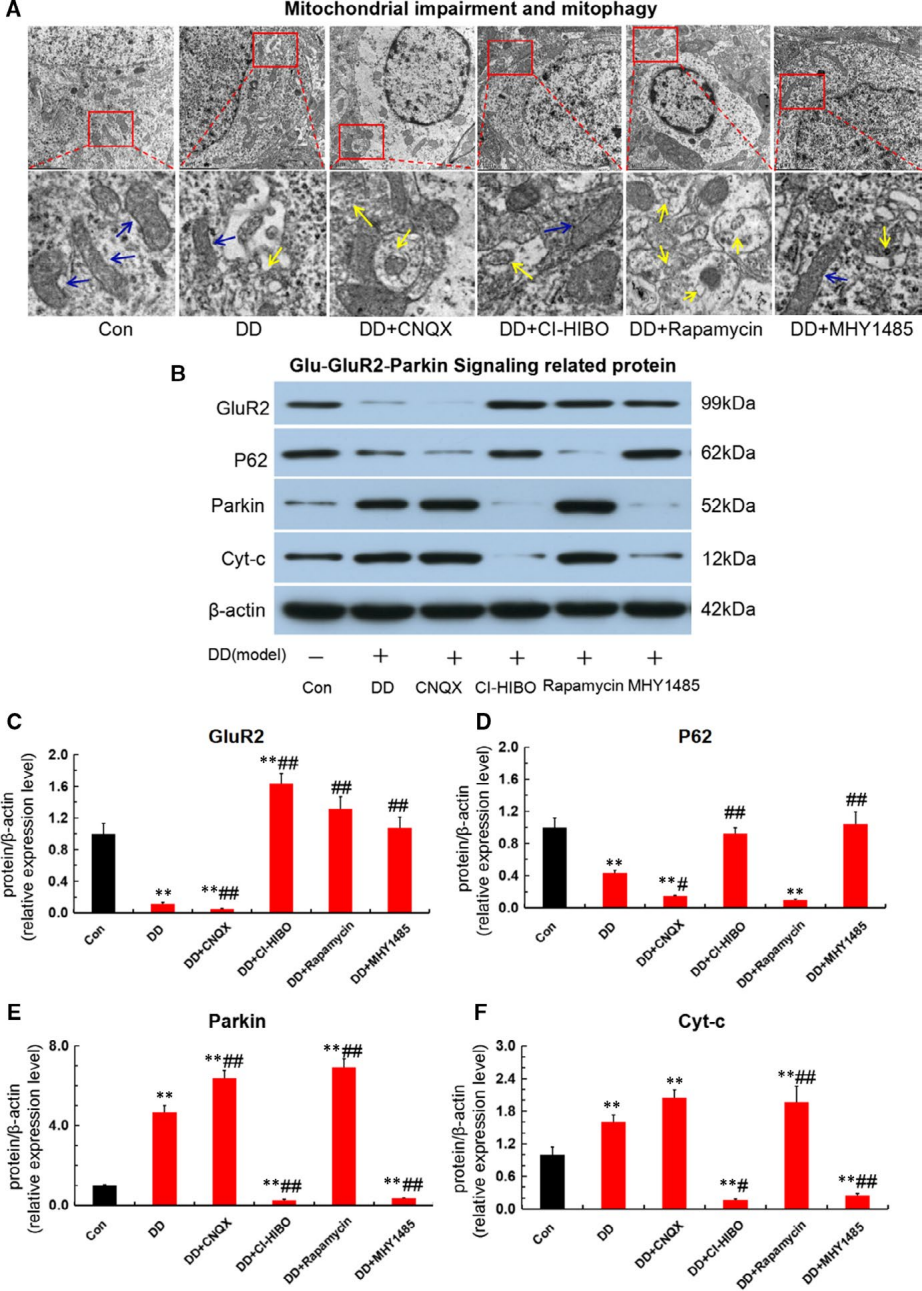
Aberrant Glu‐GluR2‐Parkin pathway triggered mitophagy‐mediated hippocampal neurons apoptosis in DD rats. (A)Mitophagy and autophagosome detected by transmission electron microscope in each group. The ‘blue arrow’ represents for the normal mitochondria and the ‘yellow arrow’ shows the mitophagy. (B‐F)Expression of Glu‐GluR2‐Parkin pathway protein and endogenous apoptotic protein for Cyt‐c detected by Western blot assay in each group (n = 3; **P < .001vs Con; ^#^ *P* < .05, ^##^ *P* < .01 vs DD)

To further confirm the depression‐like behaviour induced by mitophagy and apoptosis was associated with Glu‐GluR2‐Parkin pathway, we also prepared DD model and used transmission electron microscope to evaluate mitophagy and autophagosome and applied Western blot assay to detect the expression of Glu‐GluR2‐Parkin pathway protein and the endogenous apoptosis protein of Cyt‐c. Notably, as shown in Figure 7A, both CNQX and rapamycin exacerbated the mitochondrial impairment and promoted autophagosome formation(yellow arrows) in hippocampus of DD rat. Meanwhile, we also observed that both CI‐HIBO and MHY1485 significantly ameliorated mitochondrial impairment and lowered autophagosome formation. Additionally, the results of Western blotting analysis show that DD modelling significantly down‐regulated GluR2 and P62 (ie SQSTM1), but up‐regulated Parkin and Cyt‐c when compared with that of control(Figure 7B‐F). Interestingly, the down‐regulation of GluR2 and P62 and the up‐regulation of Parkin and Cyt‐c could be aggravated by both CNQX and rapamycin treatment when compared with the DD model group. Nevertheless, both CI‐HIBO and MHY1485 reversed the disordered expression patterns of these proteins. In brief, these results suggested that aberrant Glu‐GluR2‐Parkin pathway triggered mitophagy‐mediated hippocampal neurons apoptosis, which was responsible for monoamine neurotransmitter deficiency and depression‐like behaviour in DD rats.

## 4 DISCUSSION

Diabetes‐related depression is a major complication of diabetes mellitus.^22^ However, the pathogenesis remains to be uncovered. The current clinical studies suggest that hypothalamic‐pituitary‐adrenal (HPA) axis disruption is responsible for the occurrence of diabetes‐related depression.^23^, ^24^ The permeability of the hippocampal blood‐brain barrier (BBB) is increased due to the hyperglycaemic state, and further excessive cortisol is released in response to the dysfunction of the HPA axis.^25^, ^26^ Moreover, elevated cortisol, after crossing the BBB, activates glucocorticoid receptors (GR) and results in a dysregulation of glutamate transporters, followed by the excessive glutamate levels in intracellular.^11^ It is by now generally accepted that glutamate is an amino acid that acts as an excitatory neurotransmitter in the hippocampus.^27^ The amount of excitatory amino acid release is taken accountability for memory and cognitive dysfunction as well as monoamine neurotransmitter deficiency.^28^ In this paper, to confirm that glutamate level is of great importance for diabetes‐related depression, we prepared rat model of DD in vivo and in vitro and found that the levels of Glu, 5‐HT and DA in hippocampus were significantly increased both in DD rats and in vitro simulated DD conditions. Moreover, DD rats exhibit an obvious depressive‐like behaviour in open‐field test and in forced swimming test. Accordingly, these results provided evidence regarding the close connection between abnormal glutamate elevation and diabetes‐related depression.

The glutamate and their destructive impacts on the hippocampus have aroused a growing attention.^29^, ^30^ Evidence manifested that the content of glutamate was remarkably raised due to the dysregulation of glutamate transporters with excitatory amino acid transporter 1 (EAAT‐1) and vesicular glutamate transporter 3 (VGLUT‐3), or the disorder of glutamate reuptake and release induced by inflammatory factor with tumour necrosis factor α (TNF‐α), interleukin‐1β (IL‐1β) and interferons (IFNs) in astrocytes.^31^, ^32^, ^33^ Specifically speaking, under the condition of hyperglycaemia, the elevated pro‐inflammatory cytokine, such as TNF‐α and macrophage migration inhibitory factor (MIF), regulates glucose metabolism during systemic inflammation.^34^ On the one hand, the content of glutamate was remarkably raised due to the dysregulation of glutamate transporters, which is induced by these cytokines in astrocytes via impaired blood‐brain barrier. On the other hand, these pro‐inflammatory cytokines affect the release of glutamate by Ca^2+^‐dependent mechanism in astrocytes.^35^ Hence, cytokines such as TNF‐α, IL‐1β and MIF lead to an abnormally increased glutamate level by affecting the glutamate reuptake and release, which explain the origin of excess glutamate. Unfortunately, the explicit way how does excessive glutamate leads to hippocampal neuron apoptosis in diabetes‐related depression remains to be unclear. It is acknowledged that GluR2, mainly distributed in the hippocampal pyramidal CA1 region, is identified as an ionic glutamate receptor subunits of AMPA.^36^ Current works show that abnormally increased levels down‐regulated the GluR2, following by activation of autophagy via GluR2/mTOR pathway.^37^, ^38^ In addition, studies showed that a RAGE‐dependent impairment in the hippocampus of hyperglycaemic WT mice, with reduced AMPA receptor expression/function and LTP deficits.^39^ Our preliminary study also uncovered that the simulated DD conditions caused mitophagy via GluR2/mTOR signal in hippocampal neurons, which implied the close connection between GluR2(glutamate AMPA receptor subunit) and diabetes‐related depression.^19^, ^20^ But the exact mechanism is in need of further exploration.

Additionally, experiments reveal that mitophagy and subsequent mitochondrial function impairment contributes to the pathophysiology of depression.^7^, ^40^ Although autophagy and apoptosis are noticeable differences in morphology, the signalling crosstalk between autophagy and apoptosis is essential for the pathogenesis of depression.^15^, ^41^ Current studies have found that enhanced mitophagy and mitochondrial dysfunction resulted in deficient ATP production, an increased mitochondrial reactive oxygen species (ROS), a release of superfluous cytochrome c (Cyt‐c), up‐regulation of Bax, caspase‐3 and caspase‐9, and further an initiation of endogenous apoptosis pathways.^42^, ^43^ Further evidence also indicated that hyperglycaemia‐induced RAGE signalling altered glutamate AMPA receptor function and expression, result in an elevated glutamate levels and an increase in cytosolic reactive oxygen species (ROS), caused hippocampal neuron apoptosis and eventually led to neurotransmitter dopamine deficiency and synaptic transmission impairment.^39^, ^44^ Hence, we proposed a new hypothesis that aberrant Glu‐GluR2‐Parkin pathway led to mitophagy‐mediated hippocampal neuron apoptosis in diabetes‐related depression.

Interestingly, in the present study, our results found that the simulated DD conditions enhanced autophagic flux and mitophagy marker protein of Parkin expression via Glu‐GluR2‐mTOR signal, following by mitochondrial dysfunction with declining mitochondrial membrane potential (MMP) and elevatory reactive oxygen species as well as lactic dehydrogenase (LDH). Moreover, Tunel staining and apoptosis‐related proteins of Cyt‐c, Bax and caspase‐9 detection results indicated that the overstimulation of mitophagy exacerbated hippocampal neurons apoptosis via the death receptor pathway of endogenous (ie endogenous or mitochondrial apoptosis pathways) in simulated DD conditions. Furthermore, GluR2 receptor blocker (CNQX) and mTOR receptor blocker (rapamycin) deteriorated the decline of MMP, the increment of mitochondrial ROS and LDH, the up‐regulation of apoptosis‐related proteins of Cyt‐c, Bax and caspase‐9. Excitingly, both GluR2 receptor agonist (CI‐HIBO) and mTOR receptor agonist (MHY1485) ameliorated the overstimulation of mitophagy, dysfunction of mitochondrial and up‐regulation of apoptotic proteins. As a consequence, these findings provided evidence for aberrant Glu‐GluR2‐Parkin pathway led to mitophagy‐mediated hippocampal neuron apoptosis in the simulated DD conditions.

To further confirm the impact of mitophagy‐mediated hippocampal neuron apoptosis, which is induced by aberrant Glu‐GluR2‐Parkin pathway, on neurotransmitter levels and depressive‐like behaviour in vivo, we treated DD rats with intracerebroventricular injection of GluR2 receptor blocker (CNQX) and its agonist (CI‐HIBO), and mTOR receptor blocker (rapamycin) and its agonist (MHY1485). As expected, CNQX and rapamycin aggravated the depressive‐like behaviour in open‐field test and forced swimming test, and deteriorated the monoamine neurotransmitter deficiency of 5‐HT and DA. In contrast, both CI‐HIBO and MHY1485 alleviated the neurotransmitter deficiency and depressive‐like behaviour in DD rats. Additionally, CNQX and rapamycin exacerbated the mitochondrial impairment, promoted autophagosome formation and down‐regulated GluR2 and P62, whereas up‐regulated Parkin and Cyt‐c. In contrast, both CI‐HIBO and MHY1485 reversed the overstimulation of mitophagy and abnormal expression of these proteins in hippocampus of DD rats. Collectively, these results further support the notion that mitophagy‐mediated hippocampal neuron apoptosis, activated by aberrant Glu‐GluR2‐Parkin pathway, is responsible for depressive‐like behaviour and monoamine neurotransmitter deficiency in DD rats.

In conclusion, our findings unveil that hippocampal neuron apoptosis was triggered by mitophagy via aberrant Glu‐GluR2‐Parkin pathway and may be involved in the pathogenesis of diabetes‐related depression. This work provides promising molecular targets and strategy for the treatment of diabetes‐related depression.

## CONFLICT OF INTEREST

All authors declare that they have no competing interests.

## AUTHOR CONTRIBUTIONS

**Jian Liu:** Formal analysis (lead); Investigation (lead); Project administration (supporting); Writing‐original draft (lead); Writing‐review & editing (equal). **Lin Liu :** Formal analysis (supporting); Investigation (equal); Methodology (supporting). **Yuan‐Shan Han:** Formal analysis (supporting); Investigation (supporting); Methodology (supporting); Writing‐original draft (supporting). **Jian Yi:** Investigation (supporting); Writing‐original draft (equal); Writing‐review & editing (supporting). **Chun Guo:** Formal analysis (supporting); Investigation (supporting); Methodology (supporting). **Hong‐Qing Zhao:** Investigation (supporting); Methodology (supporting). **Jia Ling:** Investigation (supporting); Methodology (supporting). **Yu‐hong Wang:** Methodology (lead); Project administration (lead); Writing‐review & editing (lead).

## Data Availability

The raw data supporting the conclusions of this manuscript will be made available by the authors, without undue reservation, to any qualified researcher.
